# Emergency contraception from the pharmacy 20 years on: a mystery shopper study

**DOI:** 10.1136/bmjsrh-2020-200648

**Published:** 2020-06-17

**Authors:** Anna Glasier, Paula Baraitser, Lisa McDaid, John Norrie, Andrew Radley, Judith M Stephenson, Claire Battison, Richard Gilson, Sharon Cameron, Trial Steering Committee, Trial steering committee, Peter Brocklehurst

**Affiliations:** 1 Obstetrics & Gynaecology, University of Edinburgh, Edinburgh, UK; 2 Department of Sexual Health, King's College London Faculty of Life Sciences and Medicine, London, UK; 3 MRC/CSO Social and Public Health Sciences Unit, University of Glasgow, Glasgow, UK; 4 Social Science Research, The University of Queensland, Brisbane, Queensland, Australia; 5 Edinburgh Clinical Trials Unit, University of Edinburgh, Edinburgh, UK; 6 Directorate of Public Health, NHS Tayside, Dundee, UK; 7 Division of Cardiovascular Medicines and Diabetes, Ninewells Hospital and Medical School, Dundee, UK; 8 Elizabeth Garrett Anderson Institute for Women’s Health, University College London, London, UK; 9 Institute for Global Health, University College London (UCL), London, UK; 10 Sexual and Reproductive Health, NHS Lothian, Edinburgh, UK

**Keywords:** contraceptive agents, female, contraceptives, postcoital, pharmaceutical services, surveys and questionnaires

## Abstract

**Background:**

Emergency contraception (EC) was approved in the UK as a pharmacy medicine for purchase without prescription in 2001. Twenty years later we conducted a study to characterise routine practice pharmacy provision of EC.

**Study design:**

Mystery shopper study of 30 pharmacies in Edinburgh, Dundee and London participating in a clinical trial of contraception after EC.

**Methods:**

Mystery shoppers, aged ≥16 years, followed a standard scenario requesting EC. After the pharmacy visit, they completed a proforma recording the duration of the consultation, where it took place, and whether advice was given to them about the importance of ongoing contraception after EC.

**Results:**

Fifty-five mystery shopper visits were conducted. The median reported duration of the consultation with the pharmacist was 6 (range 1–18) min. Consultations took place in a private room in 34 cases (62%) and at the shop counter in the remainder. In 27 cases (49%) women received advice about ongoing contraception. Eleven women (20%) left the pharmacy without EC due to lack of supplies or of a trained pharmacist. Most women were generally positive about the consultation.

**Conclusions:**

While availability of EC from UK pharmacies has undoubtedly improved access, the necessity to have a consultation, however helpful, with a pharmacist introduces delays and around one in five of our mystery shoppers left without getting EC. Consultations in private are not always possible and little advice is given about ongoing contraception. It is time to make EC available without a pharmacy consultation.

**Key messages**

- Although availability of emergency contraception (EC) from pharmacies improves access, women in the UK still require a consultation with a pharmacist.
- One in five women may not get EC at the first visit to the pharmacy and advice about ongoing contraception is not always provided.
- Opportunities to prevent unintended pregnancy are being missed. It is time to make EC available as a general sales medicine.

## Introduction

It is more than 20 years since emergency contraception (EC) was approved in the UK as a pharmacy medicine for purchase without a doctor’s prescription. Following de-regulation, a shift to over-the-counter access occurred quite rapidly,^1^ despite EC being available free of charge from general practitioners (GPs) and family planning clinics but at a cost of around £25 in pharmacies. That shift continued with time. While 27% of women obtained their EC from community pharmacies in 2003–2004 in Great Britain, by 2007–2008 the figure had risen to 51%.^2 3^ In late 2008, levonorgestrel EC (LNG-EC, Levonelle 1500, Schering Health, UK) was made available free of charge without a prescription from pharmacies for women aged 13 years and older throughout Scotland,^4^ and Wales followed suit in 2011.^5^ In England, local arrangements allow EC free of charge from pharmacies who agree to participate in the scheme but only for certain age groups. In most pharmacies EC is provided through a ‘patient group direction’, a locally agreed proforma which lists eligibility criteria for the medicine. Although current figures are not available, it seems likely that most women now obtain EC from pharmacies rather than from a GP or a sexual and reproductive health (SRH) service, and many pharmacies in England and Wales now offer an online service with ‘click and collect’ options or even home delivery of supplies.

In 2007 we undertook a mystery shopper study^4^ to evaluate the quality of service provision in community pharmacies in Edinburgh, Scotland and to determine what advice was being given about contraception after EC use. The quality of consultations was generally good; over 75% of women were asked appropriate questions about eligibility, and over 90% received appropriate advice about how to use EC, but fewer than half the women received any advice about subsequent contraceptives. In 2018–2019 the opportunity arose to repeat the mystery shopper study in preparation for a pragmatic cluster randomised controlled trial (‘Bridge-It’ study^6^) conducted in community pharmacies in which women were offered a supply of ongoing hormonal contraception when they presented for EC, if the pharmacy was in the intervention arm of the study. The mystery shopper visits were designed to characterise ‘standard care’ in the control arm of the study, that is, the routine practice in participating pharmacies in managing requests for EC. In 2018–2019, our interest focused once again on advice about ongoing contraception after EC but also generated data on how easy (or difficult) it is to access EC in pharmacies.

## Methods

The study was undertaken in three UK cities (Edinburgh, Dundee and London) see^6^ for details. In the control arm of the ‘Bridge -it’ study LNG- EC was provided according to the pharmacies’ normal practice and women were advised to see their usual contraceptive provider for ongoing contraception. Pharmacies were trained in the conduct of the study, with training focused on provision of the progestogen-only pill not the EC. They were told that one or two mystery shopper visits would take place before the control arm of the study started, but were not told when the visits would happen. Visits took place between April 2018 and January 2019. Mystery shoppers were female volunteers aged 16 years and older who received £20 for each completed visit. Research nurses instructed them on the aims of the study, the scenario to be followed and the data to be collected. Mystery shoppers were advised that they should not swallow the EC tablet, but if pressured to do so by the pharmacist, then they should admit to being a mystery shopper. A simple scenario relating to request for EC (one episode of unprotected sex within the last 72 hours) was adapted from that used in the earlier study.^4^ Both the clinical scenario used (online supplementary table 1) and the age of the shoppers were deliberately chosen so that none were ineligible to use EC. Immediately after leaving the pharmacy, the mystery shopper completed a data collection proforma, recording the time of entering and leaving the store, the duration of the consultation and where it took place. The shopper also noted whether the pharmacist checked that they were eligible for EC and whether the information provided included specified topics such as advice on continuing contraception. These topics were included in the proforma as they constituted recommendations for the provision of EC in the national guidelines of the Faculty of Sexual and Reproductive Healthcare (FSRH).^7^ Mainly comprising tick box answers, the proforma included space for brief free-text comments relating to the experience of obtaining EC. Mystery shoppers were not given specific instructions on requesting privacy. Data from the proforma were entered into an Excel database. Two of the investigators (AG and SC) independently grouped the free-text comments of the mystery shoppers into common themes.

10.1136/bmjsrh-2020-200648.supp1 Supplementary data

### Patient and public involvement

Members of the patient and public involvement (PPI) group for the study assisted in identifying suitable mystery shoppers, and the scenario to be used as their request for EC. The PPI group are involved in the dissemination of the study results.

Ethics approval was received from South East Scotland REC in June 2017. Approvals were obtained from NHS Research Scotland (NRS) and Health Research Authority (HRA) England prior to commencement of the study.

## Results

A total of 55 mystery shopper visits (23 in Scotland and 32 in London) were conducted at the 30 study pharmacies. The mean total time (range) reported spent in the pharmacy was 12 (range 1–47) min. The median reported duration of the consultation with the pharmacist was 6 (range 1–18) min. Six mystery shoppers spent over 20 min in the pharmacy while five were there for 35 min or longer. Two complained about the long wait in the free-text comments. Consultations took place in a private room in 34 cases (62%), and the remainder at the counter, although for three mystery shoppers the consultation later moved to a private room. Not all were given EC and getting it was not always straightforward. Eight were told they had to swallow the tablet on site. One insisted that she wanted to take the EC at home and was asked to sign a form documenting this before EC was given; another was told that if she paid for EC she could take it away, but if she chose the free medicine it had to be taken on site. Four mystery shoppers were told EC was not in stock, two were 16 years old and visited the same pharmacy on the same afternoon, both were told that they should return 90 min later. Seven mystery shoppers were told that no trained pharmacist was available to provide EC, and two were persuaded that a copper intrauterine device (Cu-IUD) would be more effective and were directed to a local SRH clinic – neither was offered EC as an interim measure. One was told that she had to be registered with a GP to get free EC. In most cases where EC could not be provided an alternative pharmacy was recommended. Most pharmacies (London sites) not participating in the English scheme allowing EC to be provided free of charge recommended other pharmacies where it was available free. In all, 11 mystery shoppers (20%) were unable to get EC from the pharmacy they visited when they first presented; a further two were advised to attend elsewhere for a Cu-IUD insertion. In London, seven shoppers were told that the pharmacy could not offer free supplies, and three were offered free EC but only if they provided proof of identity and age.

Forty-eight shoppers wrote brief free-text comments about their experience in the pharmacy. The four most common themes (box 1) were the overall impression of the consultation; being told to take the EC on the premises; EC unavailable at the time of presentation; and concerns about privacy. Eighteen mystery shoppers commented very positively about the manner of the pharmacists. Positive comments about the consultations included: very informative, lovely manner, non-judgemental and understanding, very approachable, friendly, calm and reassuring. Only six mystery shoppers were at all negative. Of these, two complained that they felt the consultation was rushed, two complained about a long wait to be seen, and two mentioned the consultation being overheard by others.

Box 1 **Free-text comments from mystery shoppers**

**Overall impression of the consultation**

“Felt comfortable talking to this pharmacist, non-judgemental approach. Happy not to be ‘grilled'. Asked good questions about if was non consensual. Also advised about STI testing.” [Mystery shopper 18]

“Consulted by a very lovely informative pharmacist who covered all the basics in terms of advice given to me - even discussed upcoming research studies in EC. Pleasant experience to what can easily be an awkward conversation.” [Mystery shopper 17]

“Very rushed, pharmacist made it clear he didn’t understand what information he had to ask/explain. Pharmacist had the form in front of him but did not read from it just asked questions regarding allergies etc. and wrote at the bottom of the form.” [Mystery shopper 26]

**Told to take emergency contraception (EC) on the premises**

“… she was also insistent that I took the medication in the pharmacy but I declined explaining that I need to take medication with food so she made me sign to say that I had refused to take the medication in front of her.” [Mystery shopper 16]

“… he did however request for me to take the pill in front of him to make sure it is for me and not someone else. He said this is required legally. I then stated that I am uncomfortable with it, apologised for any inconvenience and left the pharmacy.” [Mystery shopper 49]

**EC unavailable at time of presentation**

“There was only a pharmacist on duty but she was not able to dispense the emergency contraception pill as she was not qualified. The person that usually does it was not in that day.” [Mystery shopper 53]

“The first pharmacist at the counter did not know if they are supplying EC so she called another one, who just asked whether I am registered at a GP and whether I have ID with me (she asked this all quite rudely!). She then gave me the form to complete and repeatedly asked from my ID and other details, which I was not comfortable with, so left.” [Mystery shopper 58]

**Concerns about privacy**

“Initially asked at counter and the female said to wait for the pharmacist. Had to repeat to pharmacist that I needed EC. Quite a few people around.” [Mystery shopper 55]

Table 1 shows the results for relevant information provided or elicited by pharmacist. Only 27 mystery shoppers (49%) received advice (verbal or written) about contraception after EC.

**Table 1:** Topics discussed at the 55 mystery shopper visits for emergency contraception

Topic	Yes	No	Blank
Reason for needing, or eligibility for, emergency contraception	43	12	
Verbal information/advice about importance of contraception after emergency contraception	27	27	1
Written information/advice about importance of contraception after emergency contraception	5	50	
Verbal information about local sexual and reproductive health clinic/general practice for contraception	30	25	
Verbal Information/advice about conducting a pregnancy test in 3 weeks	22	32	1
Written information/advice about conducting a pregnancy test in 3 weeks	2	53	

Blank indicates not completed.

## Discussion

This mystery shopper study suggests that getting EC from UK pharmacies is often not easy. A significant proportion of shoppers left without EC. Although some were told to return later and others were directed to a different pharmacy or to a clinic, it is possible that in real life many women would have given up the quest. Even if they did eventually manage to get a supply, the sooner after intercourse EC is taken the more likely it is to be effective, so delays jeopardise successful prevention of pregnancy. While it is heartening to learn that some pharmacists encouraged Cu-IUD insertion for EC, the mystery shoppers received verbal advice about contraception after EC in just under half of the visits, which is similar to the findings of a 2017 study.^4^ The study also demonstrated that privacy for the consultation is not guaranteed and that pharmacies sometimes request proof of identity or ingestion of EC on the premises, which are not recognised requirements in the UK.

The main limitation of this study is its size, with just 55 visits to 30 pharmacies in three different cities, and the fact that it did not involve a random sample of pharmacies (unlike our earlier study^4^) and so the findings may well not be representative. However, since all participating pharmacies had agreed to take part in, and had recently undergone training for, the Bridge-it study^6^ it is not unreasonable to assume that the performance of these pharmacies, sensitised to the EC consultation, would likely be better than average. Other mystery shopper studies from the UK have shown a similar, or worse, picture. The British Pregnancy Advisory Service (BPAS) undertaking a similar study in 30 pharmacies in England^8^ reported that while the pharmacists provided a kind and non-judgemental service, the consultation was considered ‘unprofessional’ in around 10% of visits (eg, the pharmacist demanding to see a negative pregnancy test before providing EC), and in 7% of cases the shopper was turned away without further help or told to come back later. BPAS concluded that in nearly one in five visits the woman would have missed the opportunity to prevent an unwanted pregnancy.^8^

Mystery shopper studies relating to EC access from pharmacies have also been published from other countries such as Australia and the USA.^9–13^ While the research question posed by each investigator was somewhat different to that of our mystery shopper study, all concluded that pharmacists’ practices are variable and not always in line with the recommendations in guidelines.

According to the Medicines and Healthcare products Regulatory Agency (MHRA), the underlying principle for classifying medicines in the UK is to "maximise timely access to effective medicines while minimising the risk of harm from inappropriate use".^14^ In the case of EC, timely access is critical since the efficacy of both oral EC products available in the UK declines with time following unprotected sexual intercourse.^15^ Pharmacy-only medicines can be bought, but only from pharmacies, and require the presence of a pharmacist, an arrangement which the MHRA considers as conferring "an intermediate level of control".^14^ Pharmacy-only medicines are not usually displayed on open shelves but rather are kept ‘behind the counter’, which means that women need to ask for them at the counter and this may put many women off trying to access EC.

The Royal Pharmaceutical Society^16^ argues that the mandatory consultation provides an important opportunity to ensure women are taking the EC within the correct time frame, to discuss other methods of contraception and other sexual and reproductive health issues such as sexually transmitted infection testing, and to address any safeguarding concerns. In our study only half of the pharmacists raised the issue of ongoing contraception. This is perhaps unsurprising, given pharmacies are often extremely busy and people attending a pharmacy expect a rapid service. The median time spent with the pharmacists by our mystery shoppers was 6 min, slightly less than the average duration of a consultation in general practice. However, even this may be unnecessary. The instruction for use of EC is simple, and most women in the UK present for EC well within the 72 hours time window.^17^ There are no absolute contraindications to the use of either of the currently available methods of oral EC.^7^ The UK FSRH clearly recommends that any small theoretical risk of inappropriate use is far outweighed by the risks associated with pregnancy.^7^

No mystery shopper study has evaluated consultations for EC within general practices or SRH clinics; and of course even with a prescription from a doctor, women may encounter similar barriers in terms of waiting times, lack of stock and of a trained pharmacist to fill the prescription. Although generally helpful and sympathetic, pharmacists fulfilling this "intermediate level of control" actually act as gatekeepers and, while they offer longer and much less restricted opening hours, this study and the BPAS study^8^ demonstrate that they are often unable to "maximise timely access". Our study and that of BPAS suggest that providing EC as a pharmacy medicine with the mandatory need to have a consultation with a pharmacist could result in a missed opportunity to prevent unintended pregnancy in at least 20% of attendances. The lack of provision of advice on ongoing contraception from the pharmacies in this study, or indeed the opportunity to be prescribed ongoing contraception by an independent prescriber pharmacist, is a wasted opportunity to improve the sexual health of women who preferentially access healthcare at their community pharmacy

Timely access to EC prevents pregnancy for many individual women who take it.^15^ In their review of the effects of making EC available free of charge in Wales, Mantzourani and colleagues concluded that access to free EC in pharmacies had contributed to reducing teenage conceptions.^5^ Black and colleagues^18^ analysed data from the second and third British National Surveys of Sexual Attitudes and Lifestyles undertaken in 1999–2001 and in 2010–2012, respectively. They reported increased use among single women, women living in less affluent areas and among women obtaining EC from pharmacies rather than other healthcare settings. The increased use was seen among women with some but not all risk factors for unintended pregnancy – women using less effective methods of contraception (a sizeable increase) and those with two or more sexual partners in the last year.

When EC was first approved as a pharmacy medicine in the UK it was predicted that women would abandon more effective methods of contraception, have more risky sex and that abortion rates would soar.^19^ Over the first 10 years after EC became available without prescription there was only a small rise in use in Britain from 2.3% to 3.6% of women having used EC in the last year.^18^ If more women with risk factors for unintended pregnancy are taking EC then perhaps pharmacy access is having a small public health benefit; however, the benefit could be much greater if EC was available on the General Sales List (GSL) and purchasable from a range of outlets. According to the MHRA, GSL is appropriate for medicines which can, with reasonable safety, be sold or supplied otherwise than by or under the supervision of a pharmacist. The term ‘with reasonable safety’ has been defined as "‘where the hazard to health, the risk of misuse, or the need to take special precautions in handling is small and where wider sale would be a convenience to the purchaser". While this mystery shopper study was undertaken in the context of providing LNG-EC, the need for EC, the practicalities of using it and the desirability of providing information about ongoing contraception are no different for the other oral EC available in the UK (ulipristal acetate, ellaOne, HRA Pharma) and we strongly suggest that both methods of EC should be available as GSL medicines.

Twenty years after EC was first approved as a pharmacy medicine in the UK it seems that there is more than enough evidence to demonstrate that it does not present a hazard to health, is not widely misused, does not need special precautions, and that more opportunities to obtain EC would very clearly be of benefit to women and possibly to public health.

## Conclusions

While availability of EC from UK pharmacies has undoubtedly improved access, the necessity to have a consultation with a pharmacist introduces delays, and around one in five of our mystery shoppers left without getting EC. It is time to make EC available without a pharmacy consultation.
