# Supplementary material for: Emergency contraception from the pharmacy 20 years on: a mystery shopper study
Source: BMJ Sex Reprod Health. 2020 Jun 17;47(1):55–60. doi: 10.1136/bmjsrh-2020-200648 (PMC7815628; doi:10.1136/bmjsrh-2020-200648)
Supplement: Supplementary data [file bmjsrh-2020-200648supp001.pdf]

Supplementary Table 1. Mystery shopper scenario

Mystery Shopper Sscenario :

Requesting EC for unprotected intercourse which had occurred the night before, with no other acts of intercourse in that cycle and the last (normal) menstrual period starting 11 days earlier.
